# Mesenchymal Stem Cell-Based Therapies: Challenges and Enhancement Strategies

**DOI:** 10.1007/s12013-025-01895-z

**Published:** 2025-09-26

**Authors:** Rebecca Shin Yee Wong, Ee Wern Tan, Bey Hing Goh

**Affiliations:** 1 Department of Medical Education, Sir Jeffrey Cheah Sunway Medical School, Faculty of Medical and Life Sciences, Sunway University, 5, Jalan Universiti, Bandar Sunway, 47500 Petaling Jaya, Selangor Malaysia; 2 Sunway Biofunctional Molecules Discovery Centre, Faculty of Medical and Life Sciences, Sunway University, 5, Jalan Universiti, Bandar Sunway, 47500 Petaling Jaya, Selangor Malaysia; 3 Faculty of Health, Australian Research Centre in Complementary and Integrative Medicine, University of Technology Sydney, Ultimo, Australia; 4 Graduate Institute of Cancer Biology and Drug Discovery, College of Medical Science and Technology, Taipei Medical University, Taipei, Taiwan

**Keywords:** Mesenchymal stem cell-based therapies, Challenges, Heterogeneity, Priming, Genetic modification, Cell-free strategies

## Abstract

Mesenchymal stem cells (MSCs) are popular therapeutic candidates among researchers. MSCs have been used in many preclinical and clinical studies and their safety profile and efficacy are well documented. Despite the many advantages of using MSCs in research and treatment, researchers face several challenges and limitations in MSC cell-based therapies. Over the years, many studies have explored enhancement strategies to improve the therapeutic effects of MSCs. These enhancement strategies range from generating MSCs from alternative sources, genetically modifying MSCs and priming or preconditioning MSCs in specific culture conditions before applying MSCs in treatment. On the other hand, cell-free therapies are emerging and gaining popularity in recent years. This review gives an overview of the therapeutic properties of MSCs and provides an in-depth discussion on the challenges and enhancement strategies in MSC cell-based therapies.

## Introduction

Mesenchymal stem cells (MSCs) are multipotent cells located in multiple tissues in the body. The discovery of MSCs in the bone marrow by Friedenstein and colleagues can be dated back to 1970s [1] while the term “mesenchymal stem cell” was first coined by Caplan in 1991 [2]. Since then, MSCs have revolutionized the field of regenerative medicine owing to their multipotent differentiation capacity and numerous therapeutic effects. Over the past few decades, MSCs have emerged as promising therapeutic candidates in various diseases. Since the 1990’s, there has been a growing number of clinical trials using MSCs derived from various sources, with bone marrow- (BM-MSCs), umbilical cord- (UC-MSCs) and adipose tissue-derived mesenchymal stem cells (AD-MSCs) being the commonest types of MSCs used [3].

Due to their ease in cell isolation and culture, ability to move toward sites of inflammation and injury, as well as ability to act as an immunomodulatory agent, MSCs are widely used in clinical trials globally. In a review by Zhuang et al., the top five disciplines involved in MSC-related clinical studies include neurology, orthopaedics, pulmonology, cardiovascular and vascular medicine, as well as gastroenterology [4]. On the other hand, the top five diseases investigated in clinical studies are osteoarthritis, COVID-19 related conditions (e.g. pneumonia and acute respiratory disease syndrome [ARDS]), ischaemic heart disease (IHD), graft-versus-host disease (GVHD) and spinal cord injury (based on data from ClinicalTrials.gov).

Compared to the use of other types of stem cells, such as embryonic stem cells (ESCs), usage of MSCs in research and therapy has several advantages. One of the benefits is that the use of MSCs has less ethical issues compared with ESCs [5]. However, the use of MSCs is not without disadvantages. One of the challenges in MSC research and therapy is the heterogeneity of MSC in various aspects ranging from donor variations to functional heterogeneity due to differences in cell culture, expansion, preservation and routes of administration [6]. Although MSCs are generally considered cells with low immunogenicity, immunocompatibility may still be a concern in cell-based therapies. For example, research has shown that repeated intra-articular injections of allogeneic MSCs in an animal model resulted in adverse clinical response, suggestive of allogeneic MSC immune recognition by the host [7].

In view of the challenges in MSC cell-based therapies, researchers have explored various ways to overcome these challenges. This review, therefore, gives an overview of MSCs and the limitations in MSC cell-based therapies, accompanied by a detailed discussion on enhancement strategies to improve therapeutic efficacy of MSCs, such as strategies to overcome MSC heterogeneity and methods to modify or prime MSCs, as well as some examples of cell-free strategies.

### Overview of MSCs and their Therapeutic Properties

MSCs are multipotent stem cells residing in many locations in the body such as the adipose tissue, bone marrow, dental pulp, placenta, amniotic fluid, umbilical cord and Wharton’s Jelly [8]. Earlier studies have shown that MSCs can exist in at least three morphological subpopulations: (1) rapidly self-renewing cells, (2) spindle-shaped cells resembling fibroblasts and (3) large, flattened cells that are slowly replicating [9]. In 2006, the International Society for Cell and Gene Therapy (ISCT) published the minimal criteria for the characterisation of MSCs, which include (1) the ability to adhere to plastic in tissue culture, (2) the presence and absence of certain surface markers and (3) the ability to differentiate into certain cell types (adipocytes, chondrocytes and osteoblasts) [10].

The definition of MSCs was recently updated recently by an international Delphi consensus panel and comprehensive reporting guidelines for MSC-based products were suggested. This consensus defined nine key defining criteria, including uniform nomenclature, expression profiles of markers, tissue of derivation, and essential attributes, including potency and viability [11]. Furthermore, 33 items were suggested among clinical studies including MSC source, culture conditions, administration protocol, and product analysis [11]. These investments are critical for consolidation and transparency in MSC research and clinical translation.

The availability of MSCs in multiple locations and the ease of harvesting are among the many reasons why these cells are very popular among researchers and clinicians. MSCs are also relatively easy to culture in the laboratory. Due to their immunosuppressive and immunomodulatory nature, the application of allogeneic and autogenic autologous MSCs have been reported in previous studies [5]. In a meta-analysis that explored the safety profile of MSCs in 62 randomized clinical trials (*n* = 3546) covering about 20 diseases, MSC administration were reported to be safe and well-tolerated among different populations, which was accompanied by some non-serious side effects such as transient fever, constipation, sleeplessness, fatigue and adverse events at the site of administration [12].

**Fig. 1:**
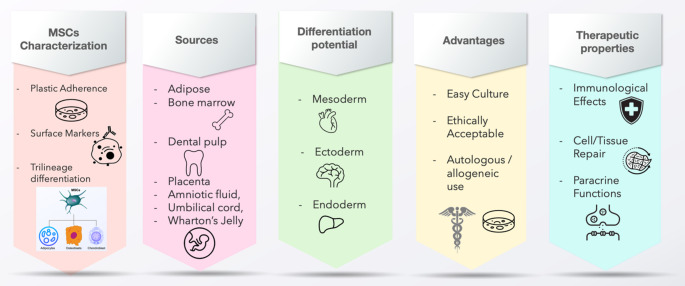
Summary of properties of MSCs

One of the therapeutic properties of MSCs is their multipotent potential. Under specific conditions, MSCs differentiate into cells of mesodermal lineage such (e.g. adipocytes, chondrocytes, osteocytes, muscle cells, endothelial cells etc.). However, studies have shown that MSCs’ differentiation potential is not limited to mesodermal derivatives. Transdifferentiation of MSCs into cells of ectodermal (e.g. neurons and hepatocytes) and endodermal (e.g. pancreatic beta islet cells) origins have also been reported [13]. Therefore, MSCs is known to have a triphoblastic differentiation potential. MSCs are capable of exerting anti-inflammatory, immunosuppressive and immunomodulatory effects, which make them good therapeutic candidates in some immune-mediated diseases [14, 15]. Other therapeutic effects of MSCs include (1) the ability to migrate to sites of injury and inflammation for repair and regeneration [16], (2) anti-apoptotic effects [17], (3) pro-angiogenic effects [18] and (4) anti-fibrotic effects [19]. The properties of MSCs are summarised in Fig. 1.

While largely attributed to much older literature, it is now well recognized that the therapeutic properties of MSCs go well beyond their capacity for differentiation into multiple cell types. Though during the early years of MSC research attention was focused on the possible engraftment of MSC into damaged tissues and the subsequent in vivo differentiation into damaged cell types, more recent studies have emphasized the fact that most of their beneficial effects are due to the secretion of bioactive factors. By releasing various cytokines, chemokines, growth factors and extracellular vesicles, MSCs are able to regulate the immune response, promoting angiogenesis, inhibiting apoptosis, and activating endogenous regeneration pathways. This secretome-mediated activity is now considered a central mechanism of action, recasting the notion of MSCs as mere building blocks for tissue healing to orchestrators of repair. Nevertheless, the ability of MSCs to differentiate or transdifferentiate in vivo in a significant and stable manner is under debate and merits an in-depth discussion.

### In Vivo Differentiation and Transdifferentiation Potential of MSCs

MSC with proved differentiation potential have been a topic of much discussion. Transplanted MSCs were able to engraft within tissues under damage, becoming mesodermal derivatives such as bone, cartilage, skeletal muscle and vascular endothelium, as revealed in early animal studies [20]. There were also reports of MSCs transdifferentiating into cells of ectodermal and endodermal origin, including neurons, hepatocytes, and pancreatic β-cells [21]. These findings initially affected optimism that MSCs might directly participate in repairing the structure of a tissue. However, much of this early evidence was based on histological markers, without serious lineage tracing, raising questions about whether the observed phenotypes represented true differentiation or instead were accidental artifacts caused by cell fusion, uptake of host cell proteins and transient morphological changes [22].

Recent studies using genetic fate mapping and advanced imaging have called into doubt the idea that MSCs can appropriately engraft and differentiate in vivo, particularly in large animal models and clinical situations [23]. Instead, it is suggested that MSC engraftment efficiency is usually low, transient with most transplanted cells being cleared within days to weeks after administration. For example, lineage tracing experiments in murine models have shown that only a small fraction of MSCs integrate into host tissue and these cells seldom retain long term [24]. Moreover, functional improvement in MSC-treated animals often occurs even when detectable engrafted donor cells are absent, suggesting that paracrine-mediated tissue repair by MSCs may be far more common than direct differentiation into target cell types [25].

In line with growing trends in this field, it is currently thought that the primary therapeutic effects of MSCs come from paracrine as opposed to structure-based integration. MSCs release an extensive repertoire of bioactive molecules, extracellular vesicles and growth factors that adjust immunological reactions, excite natural progenitor cells, promote angiogenesis/ tissue remodeling [26]. This change of perspective has significant implications for clinical translation. While the interest lies in strategies to improve MSC engraftment and differentiation, more emphasis is now placed upon optimizing the composition of their secretome, the regimens in which they are preconditioned and how they are delivered so as to maximize these paracrine-mediated benefits. Yet, underpinning the precise conditions under which MSCs can differentiate or transdifferentiate in vivo remain relevant, particularly for regenerative medicine protocols needing structural tissue replacement.

## Therapeutic Applications of MSCs

MSCs have attracted interest for their regenerative, immune modulatory, anti-inflammatory, and trophic effects. The cells are easy to isolate, poorly immunogenic and have a homing to sites of injury which renders them attractive candidates for a number of therapeutic indications [27]. Its therapeutic promise for tissue repair, immune modulation, and treatment of degenerative and inflammatory diseases has been explored in hundreds of preclinical and clinical studies in the last two decades [28]. The following sections provide a general overview of the major therapeutic categories in which MSCs have shown promising outcomes.

### MSCs in Regenerative Medicine

The regeneration of damaged or diseased tissues by MSCs has been suggested to be the result of both differentiation of MSC’s into target cells and paracrine signaling where they have been studied extensively in orthopaedics for repair of bone, cartilage and tendon injuries [29]. For example, MSC autologous or allogeneic transplantation has been applied in clinical trials for osteoarthritis and bone nonunion [30]. MSCs have also been used in the area of cardiovascular sciences, where they may contribute to neovascularization and improve cardiac function following myocardial infarction [31]. In neurodegenerative diseases including spinal cord injury and stroke, MSCs could potentially contribute to neuronal repair by releasing neurotrophic factors and anti-apoptotic molecules, albeit successful functional integration [32].

Paracrine activity of MSC such as extracellular vesicles (EVs), GFs or cytokines release is acknowledged to be a particurlarly important player in reprogramming the regenerative outcome, where they released factors mediate the local microenvironment and stimulate the endogenous progenitor cells homing and decreasing apoptosis, inflammation, and fibrosis at the site of injury [33]. In respect to this, MSCs function more as “cellular drug factories” rather than as bricks-and-mortar for tissue.

### MSCs in Immune and Inflammatory Disorders

In addition to their effects on tissue regeneration, MSCs have now been recognised as powerful chemomodulators by suppressing immune cell proliferation, shifting cytokine profiles and inducing immune tolerance [34]. These have rendered MSCs an appealing option for treating autoimmune and inflammatory diseases. For instance, MSCs have been applied to the therapy of GvHD, focusing on steroid-refractory GvHD, and remestemcel-L have are already commercially approved in some parts of the world [35]. In autoimmune disorders, including systemic lupus erythematosus, multiple sclerosis and Crohn’s disease, MSC treatment appears to have the potential of decreasing disease activity and immunomodulation [36]. MSCs have also been investigated in respiratory diseases, including acute respiratory distress syndrome (ARDS) including COVID-19 associated ARDS, due to their anti-inflammatory and regenerative properties [37]. These systemic immunomodulatory effects highlight the potential of MSCs for treatment and justify their ongoing clinical trial evaluation across a range of inflammatory and immune diseases.

### Clinical Applications and Translational Progress

A number of clinical trials have now tested the safety and efficacy of MSCs in a wide range of indications, from orthopaedic disorders and cardiovascular diseases to immune mediated diseases. The safety of MSCs has been uniformly good and very few adverse events have been reported [12]. A few MSC-based products have already been commercialized in some countries, such as Prochymal (for GvHD) and Cartistem (for knee cartilage defects), indicating the clinical feasibility of MSC therapies [38]. Despite these advancements, the clinical application of MSC-based therapies is still restricted worldwide. Disparities in the clinical outcomes of trials, heterogeneous cell types and production process variations, and failure to achieve consensus on therapeutic endpoints explain the mixed clinical outcomes. These translational obstacles emphasize the necessity to recognize and meet the biological and technical challenges that result in restricting the broader utilization of MSCs therapy, and will be further reviewed in the following section.

### Emerging Cell-Free MSC Therapies

More recently, research attention has been shifted not too much towards the cells themselves but the paracrine factors MSCs secrete, predominantly EVs and exosomes. These cell-free products contain proteins, lipids and RNAs, which are responsible for many the therapeutic effects of MSCs, such as immune modulation and tissue repair [39]. The use of cell-free therapy has several advantages over live-cell transplantation, including low immunogenicity, simple storage, and better safety [40]. Nonetheless, the therapeutic use of MSC-secretomes is currently being explored. It is difficult to isolate, standardize and scale these products and to elucidate the exact mechanisms by which they mediate responses [41]. Despite the promise, cell-free MSC strategies experience similar regulations, manufacturability, and efficacy issues that stall whole cell therapeutics. These constraints, as well as the constraints encountered in cell-based MSCs utilization, are reviewed in the next section of this chapter, which is about the challenge in MSC therapy.

## Challenges in MSC cell-based Therapy

Despite the many advantages of using MSCs and their numerous beneficial effects, limitations and challenges exist for the use of MSCs in research and treatment. These limitations occur at multiple levels throughout the bench-to-bedside process, as challenges may arise anywhere from the bioprocessing to the administration of MSCs.

### Heterogeneity of MSCs

The heterogeneity of MSCs is a common challenge to researchers and clinicians. Heterogeneity exists in various aspects, which leads to phenotypical and functional variations. These variations, in turn, lead to difficulty in the development of standardised procedures in cell processing and applications, such as isolation, culture, expansion, differentiation, preservation, release and administration of MSCs. This section discusses various factors influencing MSC heterogeneity.

#### Donor Factors

Factors such as the age, gender and health status of the donor contribute to functional heterogeneity in MSCs. For example, Yamaguchi et al. compared the therapeutic effects of human MSCs (hMSCs) from young and old donors in a rat model of ischaemic stroke [42]. Rats transplanted with young hMSCs had better behaviour recovery with brain atrophy prevention, when compared with rats receiving old hMSCs. Treatment with young hMSCs also resulted in less microglia, more blood vessels, as well as more pronounced neural stem/progenitor cell migration in the cortex surrounding the area of infarct, when compared with the use of old hMSCs. These findings suggest that age plays a role in the therapeutic effects of MSCs, with young hMSCs giving a better functional recovery due to vessel maturation, neurogenesis and anti-inflammatory effects.

Another study demonstrated that female MSCs were more efficacious than male MSCs in the treatment of neonatal hyperoxia-induced lung injury. Rats exposed to hyperoxia showed improvements in vascular remodelling and lung inflammation after administration of male or female MSCs. However, a greater therapeutic efficacy was observed in rats administered female BM-MSCs. Interestingly, the therapeutic efficacy of female MSCs was more pronounced in male rats. These findings suggest that gender may play a role in MSC therapeutic efficacy for lung repair in bronchopulmonary dysplasia complicated by pulmonary hypertension [43].

Other than age and gender, the health status of the donor may also contribute to the functional heterogeneity of MSCs. Nguyen et al. studied the effects of disease duration and obesity on the efficacy of MSC treatment in type 2 diabetes mellitus (T2DM) patients using autologous BM-MSCs [44]. The treatment was efficacious in patients who had T2DM for < 10 years and a body mass index (BMI) of < 23. There was a direct correlation between the duration of T2DM and altered MSC proliferation rate, as well as accumulation of mitochondrial DNA mutations. Disease duration was also associated with abrogation of mitochondrial respiration and glycolysis in BM-MSCs. Therefore, the study concluded that BM-MSC treatment for T2DM should be performed in non-obese diabetic patients with a disease duration of < 10 years, as the health status of the patient has an influence on treatment efficacy of IV BM-MSC transplantation in T2DM.

Kim et al. compared several properties of MSCs from patients with cerebellar ataxia (CA-MSCs) and those from healthy subjects (H-MSCs) [45]. Through an analysis of the secretome, CA-MSCs showed reduced capacities in (1) motility, (2) proliferation, (3) immunomodulatory functions and (4) response to oxidative stress when compared to H-MSCs. The functional discrepancies were validated via neuroglia co-cultures. Further exploration revealed that follistatin-like 1 (FSTL1), an immunoregulatory and neuroprotective protein was downregulated. The findings suggest that MSCs from patients and healthy subjects exhibit functional variations. The study, therefore, concluded that strategies that target the related aspects of the secretome may help enhance the efficacy of treatment using autologous MSCs.

#### Source-Dependent Variability in MSC Characteristics

It is now clear that MSCs from different sources exhibit differences in terms of their differentiation potential and other functional characteristics. In the published literature, information on the variations in MSCs from different sources is overwhelming. Table 1 gives some examples of differences between MSCs from different sources, with a focus on studies in the past 5 years.

**Table 1:** Summary of differences between MSCs from different sources

Source of MSCs	Key findings	Reference
• PAT-MSCs • LAT-MSCs	• PAT-MSCs had a significantly higher osteogenic potential thatn LAT-MSCs • Alkaline phosphatase activity was significantly higher in PAT-MSCs than LAT-MSCs. • PAT-MSCs and LAT MSCs demonstrated differential expression in 255 gene sequences	[88]
• Foetal MSCs from umbilical cord, amniotic membrane, and chorionic plate. • Maternal MSCs from decidua parietalis	• Both foetal and maternal MSCs showed similar multipotent potential and phenotype • Foetal MSCs demonstrated a higher expansion capacity • Different levels of paracrine factors were observed in MSCs from all sources	[89]
• AT-MSCs • BM-MSCs • UC-MSCs	• Comparing MSCs from different sources in the treatment of GVHD. In vitro: • AT-MSCs and BM-MSCs were more potent in suppression of lymphocyte proliferation. • AT-MSCs and UC-MSCs induced a higher regulatory T cell/ T helper cell ratio. • AT-MSCs and UC-MSCs more potent in coagulation pathway activation than BM-MSCs. In vivo: • In GVHD mouse model, AT-MSCs, BM-MSCs and UC-MSCs failed to significantly increase overall survival rate or prevented death from GVHD.	[90]
• UCB-MSCs • UCT-MSCs	• Differences in key surface markers of MSC (CD90 and CD 105) reported. • UCT-MSCs had several advantages over UCB-MSCs in terms of yield, purity, culture time, rate of senescence in vitro. • UCT-MSCs showed more robust myogenic differentiation	[91]
• AD-MSCs • BM-MSCs	• Meta-analysis on effect of MSCs on osteoarthritis; 19 studies; *n* = 811 • At 6 months: AD-MSCs showed better VAS and WOMAC than BM-MSCs • At 1 year: AD-MSCs shower better WOMAC, KOOS and WORMS than BM-MSCs • At 24 months: AD-MSCs showed better Lysholm score than BM-MSCs but BM-MSCs showed better VAS	[92]
• rDPSCs • rBM-MSCs	• Both rDPSCs and rBM-MSCs could induce osteogenesis in rats with mandibular congenital defects. • rDPSCs was a better source as both undifferentiated and differentiated cells promoted bone regeneration, whereas for rBM-MSCs, only differentiated cells were involved in bone defect repair.	[93]
• BM-MSCs • aBM-MSCs	• Comparison between BM-MSCs and aBM-MSCs from 6 patients. • Transcriptomic analysis showed substantial gene expression variations (589 genes) between BMSCs and aBMSCs. • aBMSCs induced greater angiogenesis and showed superior bone regenerative abilities compared to BMSCs.	[94]
• LB-MSCs • M-MSCs	• In vitro and animal studies showed that M-MSCs had a higher rate of proliferation and greater osteogenic potential when compared with LB-MSCs.	[92]
• AD-MSCs • BM-MSCs • L-MSCs	• Comparing AD-MSCs, BM-MSCs and L-MSCs from COPD patients and non-COPD controls at the transcriptome level. • MSCs from various sources exhibited distinct gene signatures, reflecting heterogeneity among donors. • All MSC sources showed altered gene expression in COPD patients compared to controls.	[95]
• iPSC-MSCs • BM-MSCs	• Compared to BM-MSCs, iPSC-MSCs showed increased proliferation and variable differentiation potential based on donor cells	[96]
• AF-MSCs • AS-MSCs • UCB-MSCs • WJ-MSCs	• AF-MSCs showed the highest growth rate • AF-MSCs had the highest colony-forming efficiency • AS-MSCs had the longest doubling time • AF-MSCs demonstrated the most robust growth and promising characteristics, outperforming MSCs from other sources.	[97]
• BM-MSCs • UC-MSCs • UCB-MSCs	• UC-MSCs outperformed BM-MSCs and UCB-MSCs in terms of differentiating into tendon-like lineage cells • UC-MSCs were found to enhance full-thickness tendon defect regeneration in rats when compared to BM-MSCs and UCB-MSCs	[98]

*aBM-MSCs* Alveolar Bone Tissue-derived MSCs, *AD-MSCs/ AT-MSCs*, Adipose-Derived/ Adipose Tissue- Derived MSCs, *AF-MSCs* Amniotic Fluid-Derived MSCs, *AS-MSCs* Amniotic Sac-Derived MSCs, *BM-MSCs* Bone Marrow-Derived MSCs, *iPSC-MSCs* Induced pluripotent stem cell-derived MSCs, *KOOS* Knee Osteoarthritis Outcome Score, *LAT-MSCs* Lipoaspirated Adipose Tissue-Derived MSCs, *L-MSCs* Lung-Derived MSCs, *LB-MSCs* Long Bone-Derived MSCs, *M-MSCs* Mandible-derived MSCs, *PAT-MSCs* Palatal Adipose Tissue- Derived MSCs, *rBM-MSCs* Rat Bone Marrow-Derived MSCs, rDPSCs, *UCB-MSCs* Umbilical Cord Blood-Derived MSCs, *UC-MSCs* Umbilical Cord-Derived MSCs, *UCT-MSCs* Umbilical Tissue Derived MSCs, *WOMAC* Western Ontario McMaster Universities Osteoarthritis Index, *Lysholm* Lysholm Knee Scale, *WORMS* Whole-Organ Magnetic Resonance Imaging Score.

#### Additional Factors Influencing MSC Heterogeneity

Other than factors related to the donor and source of MSCs, heterogeneity in MSCs may be contributed by factors related to culture conditions, route of administration, health status of the recipient etc. Table 2 summarised selected studies on non-donor related factors contributing to MSC heterogeneity.

**Table 2:** Non-donor related factors contributing to heterogeneity of MSCs

Factor	MSC type	Key findings	Reference
Clonal variability	BM-MSCs	• Subpopulations of BM-MSCs with a difference in potency existed, with CD146 as a marker of multipotency. • Average colony-forming efficiency (CFE) of 55–62% was observed. • Tripotent MSCs consisted of 50% of colony-forming cells • Tripotent clones demonstrated better CFE, cell amplification and colony diameter when compared to unipotent clones.	[99]
	UC-MSCs	• Heterogeneity was due to three distinct clusters (C1, C2, and C3) of UC-MSCs with different expression of cytokines, stemness markers. And immunosuppressive activities.	[100]
	WJ-MSCs	• Heterogeneity was due to the presence of distinct subpopulations. • Subpopulations of WJ-MSCs have different characteristics such as proliferation, niche support, metabolism, and bio-functional properties.	[101]
Culture conditions (surface geometry)	hBM-MSCs	• Migration speed of hMSCs was faster on concave spherical surface than flat and convex spherical surfaces. • Convex spherical surface led to substantial deformation of the nucleus and promoted osteogenic differentiation.	[102]
Culture conditions (composition)	AT-MSCs	• Higher proliferation rate was observed in MSCs cultured in medium free from antigens derived from animals (xeno free) than MSCs cultured in xenogeneic medium.	[103]
Culture conditions (composition)	hBM-MSCs	• Used of various growth factors to investigate cell proliferation and osteogenic differentiation of hBM-MSCs. • Enhanced cell proliferation with the use of TGF-β and bFGF in culture medium. • Enhanced osteogenic differentiation with the use of VEGF and IGF-1 in culture medium.	[104]
Harvest methods	AD-MSCs	• Higher proliferation and better resistance to senescence observed in MSCs obtained through power-assisted liposuction than MSCs obtained through surgical biopsy and laser-assisted liposuction.	[105]
Route of administration	MSCs of various sources	• Systematic review of 109 articles concerning effects of route of administration on biodistribution. • IV: MScs trapped in lungs and later redistributed to spleen, liver and kidneys • IA: MSCs bypassed the lungs and widely distributed throughout the body • IM, IArt and ID: Localised and lack of systemic biodistribution	[106]

*AD-MSCs/AT-MSCs* Adipose-Derived/ Adipose Tissue- Derived MSCs, *bFGF* Basic Fibroblast Growth Factor, *hBM-MSCs* Human Bone Marrow-Derived MSCs, *IA* Intraarterial, *IArt* Intra-articular, *ID* Intradermal, IGF = 1 = Insulin Growth Factor-1, *IM* Intramuscular, *IV* Intravenous, *TGF-β* Transforming Growth Factor Beta, *VEGF* Vascular Endothelial Growth Factor

### Replicative Senescence and Differentiation Constraints of MSCs

The ability of MSCs to replicate and live in cell cultures is not perpetual. Research has shown that when cultured in the laboratory, MSCs become senescent due to in vitro aging. Early research showed that MSCs had a mean long-term culture of 118 days and a mean average passage number of eight. The population doubling (PD) and telomere length (TL) decreased with increasing passage number. At the 10th passage, PD dropped from 7.7 to 1.2, while at the 9th passage the TL reduced from 9.19 to 8.75 kbp. Every two passages resulted in 100 bp of telomere shortening. A reduction in differentiation potential to osteocytes and adipocytes was also observed during late passages [46].

Another study showed that after several passages, MSCs demonstrated heterogeneity in clonal expansion and colony formation. The number of colony-forming unit (CFU) fibroblasts significantly decreased with increasing passage number, and was hardly detectable after > 20 passages., In addition, after a few passages, the number of MSC subpopulations with high differentiation potential was greatly reduced [47]. These findings implicate that the use of MSCs for therapy should be considered early on rather than late, as the cells lose their stemness and ability to proliferate as they age.

Although MSCs are well known for their triplobastic differentiation potential, research has shown that the efficiency of MSCs differentiating into ectodermnal, mesodermal and endodermatl derivatives is limited. For instance, only about 3% of MSCs were reported to differentiate into neurons (ectodermal) [48], whereas about 5% of MSCs can differentiate into insulin positive pancreatic beta-islet cells (endodermal) [49]. On the other hand, about 10–25% of MSCs can differentiate into osteocytes (mesodermal) [50]. These limitations in lifespan, clonal expansion and differentiation potential have clinical significance, as MSCs with a large pool of senescent cells are not likely to be efficient when transplanted because senescent MSCs exhibit phenotypic and functional alterations.

Interestingly, Leng et al. showed that even MSCs from the same source may exhibit differences in stemness and differentiation potential [51]. The study showed that MSCs from Wharton jelly (WJ-MSCs) highly expressed genes associated with mesodermal differentiation, but partially expressed genes associated with endodermal differentiation and hardly expressed genes associated with ectodermal differentiation. The potency of WJ-MSCs also differed among different subpopulations from the same source, i.e., some are bipotent while others are unipotent. The study concluded that only a subpopulation of WJ-MSCs were identified to be true stem cells and these cells were not immune privileged cells.

#### Paracrine Mechanisms as a Central Challenge

Although MSCs were first proposed as promising candidates by virtue of their ability to engraft and undergo differentiation into tissue-specific cell lineages, there is now emerging evidence to show that the therapeutic benefits of these cells are predominantly mediated through paracrine actions rather than engraftment of cells in vivo in a stable differentiated state. The MSC secretome, which is composed of cytokines, chemokines, growth factors, and EVs, is emerging as the critical mediator of immunoregulation, angiogenesis, anti-apoptosis, and induction of endogenous regenerative mechanisms [52]. This change in perception reframes the obstacles in MSC-based therapies, away from simply increasing differentiation capacity toward the knowledge that donor variance, replicative senescence, and culture parameters contribute to the repertoire and capacity of the secretome.

A unique difficulty is the variable secretome composition between MSC sources and culture conditions, thus complicating standardization and reproducibility. For instance, hypoxic pre-conditioning/inflammatory priming markedly skewed the content of MSC-EVs and correspondingly influenced their immunosuppressive or pro-regenerative properties [53]. Similarly, senescent MSCs could secret a secretome that is pro-inflammatory, which could limit therapeutic potency if not result harmful [54]. These results indicate that secretome reproducibility and consistency is as important as cell viability or differentiation, especially for clinical translation.

The possible contribution of other modes of action as well as paracrine effects mediated via exosomes may be also relevant to circumvent the drawbacks of cell-based strategies. Breaking the code for being able to control and standardize secretome activity could help us solve the problem of poor engraftment, heterogeneity and short lifetime of implanted cells. Furthermore, this concept may offer a conceptual foundation for the application of cell-free based therapies, in which purified secretome components or MSC derived EVs may outstrip numerous inherent restrictions of living cell therapies while maintaining their therapeutic effectiveness.

### Additional Challenges in MSC-Based Therapies

One of the challenges in the bioprocessing of MSCs is the large-scale production of MSCs that mimic cells of natural niches. This is often difficult due to the heterogeneity of MSCs and a lack of standardised protocols at various levels of bioprocessing. The upstream processing (USP) may take one of the two approaches, namely autologous (one to one) and allogeneic (one to many) approaches. The key differences between the two approaches are the number of therapeutic doses produced and the number of patients benefias well as approval processesting from the MSCs generated. The former produces multiple small batches with each batch yielding a few therapeutic doses for one patient, whereas the latter generates one large batch yielding multiple doses for many patients. From an economic viewpoint, the allogeneic approach for MSC therapy is preferred commercially, because autologous manufacturing incurs a higher cost and is subject to stricter security and quality control [55].

USP of MSCs includes cell isolation, expansion and harvest. The culture media used in USP need to be supplemented with various expensive additives. Conventionally, foetal bovine serum (FBS) is used in MSC culture, as it provides the adhesive proteins, hormones and growth factors required for cell maintenance and proliferation [56]. Although FBS has been used in clinical application of MSCs [57], xeno-free media (XFM) are preferred as xenogeneic media possess the risks of passing animal factors to patients and elicitation of host immune response, especially after repeated administrations [58]. Various in-house and commercial XFM have been reported in the published literature. It is noteworthy that the influences of these XFM are inconsistent among studies and are dependent of the type of MSCs used. Therefore, medium testing for suitability of XFM for different types of MSCs is necessary [59].

On top of the choice of medium, other factors to be considered in USP and downstream processing (DSP) of MSCs include (1) choice of microcarrier, (2) choice of reactor system, (3) methods used in cell harvest (e.g., choice of enzymes and detachment conditions, 4) methods used in cell separation, clarification and concentration, 5) formulation strategies and storage methods, and 6) quality approval (including identity, purity, potency and viability of MSCs). As every step along the way influences the quality of the end product, strict adherence to good manufacturing practice (GMP) is important. Therefore, the challenges are many, from skills of the personnel handling the process to cost efficiency, as well as the fulfilment of cell quality characteristics required for clinical application [60].

Other challenges in MSC cell-based therapies include gaps in standardized characterisation guidelines and regulation of clinical use of MSC-based products. To date, at least 10 MSC-based products (consisting of autologous and allogeneic MSCs from various tissue sources) have received regulatory approval in various parts of the world such as South Korea, Europe, Canada, New Zealand, Japan etc [55, 61]. In December 2024, the US Food and Drug Administration (FDA) approved Mesoblast’s Ryoncil (remestemcel-L-rknd), an allogeneic bone marrow-derived mesenchymal stromal cell (MSC(M)) therapy, marking the first FDA-approved MSC-based product for clinical use in the United States [62]. In some countries, stem cell therapy is regulated by law, whereas in others, there is no law to regulate stem cell therapy [63]. Therefore, there remain huge regulatory gaps in MSC therapy. To makes matters even more challenging, each country has its own unique sets of rules and regulation, as well as approval processes.

## Enhancement strategies in cell-based therapies

Researchers have explored various enhancement strategies to overcome the challenges faced in cell-based therapies. This section discusses different approaches in combatting the limitations in cell-based therapies. In general, these strategies belong to three main categories, namely (1) strategies to overcome MSC heterogeneity, (2) strategies to enhance the biological functions of MSCs to achieve the desired therapeutic effects and (3) cell-free strategies.

### Overcoming MSC heterogeneity

It is now clear that MSC heterogeneity are multifactorial, occurring at different levels of the bench-to-bedside process. Some factors (e.g. donor’s age, gender, health status) are non-modifiable. On other hand, modification of other factors (e.g. sources and bioprocessing of MSCs) is possible. To this end, researchers have turned their attention to generating MSCs from induced pluripotent stem cells (iPSCs). iPSCs are cells generated from adult somatic cells (e.g. skin cells). The production of iPSCs involves genetic reprogramming of somatic cells, with forced expression of certain genes and factors. Lpotential of primary MSCs, as it is possible ike embryonic stem cells (ESCs), iPSCs show pluripotency, express markers of ESCs and share many functional properties of ESCs [64] and they are not associated with the ethical concerns of ESCs.

In an earlier study, Hynes et al. demonstrated the generation of MSCs from iPSC cell lines of several somatic cell types (i.e. lung, gingiva and periodontal ligament) [65]. The iPSC-derived MSCs (iMSCs) were functional and shared many morphological and functional characteristics of primary MSCs derived from tissues [66]. For example, iMSCs displayed the typical fibroblastic morphology of primary MSCs, expressed several markers associated with MSCs and exhibited the ability to differentiate into adipocytes, chondrocytes and osteoblasts [67, 68]. Other similar studies have also reported the production of iMSCs from iPSCs using different methods [69].

There are many advantages of iMSCs over primary MSCs. Firstly, the unlimited source of iPCSs help to overcome the issue of limited patient samples, especially when multiple injections require a large number of autologous cells [70]. Secondly, compared to the high heterogeneity of primary MSCs, iMSCs are more homogenous and stable in quality, because generation of iMSCs from a single clone of iPSCs is possible [71]. Thirdly, scalable production at reasonable costs is feasible, without the need to obtain primary cells from the patients repeatedly [22]. Lastly, the use of iPSCs overcome the issues of limited lifespan, expansion and differentiation potential of primary MSCs, as it is possible to generate iMSCs from infinite iPSCs [71].

### Strategies to Enhance MSC Therapeutic Potency

Research has shown high variations in the therapeutic effects of MSCs due to heterogeneity and other factors. Enhancement of the biological functions of MSCs for better treatment efficacy involves two main approaches, namely genetic modifications of MSCs and preconditioning or “priming” of MSCs.

#### Genetic modification of MSCs

Genetic modifications of MSCs is feasible using non-viral or viral vector methods. The use of a viral vector as a tool for gene transfer is common due to its high efficiency. Some examples of viral vectors include adenoviral, lentiviral, retroviral, adeno-associated virus based and other viral vectors [72, 73]. However, the use of viral vectors is associated with several disadvantages such as high costs for cell line production, elicitation of adverse host immune response, as well as insertional mutagenesis, which may lead to oncogenesis [74]

Non-viral methods of genetic modification of MSCs include chemical and physical approaches. Examples of physical methods include electroporation, sonoporation and nucleofection, whereas chemical methods include the use of inorganic nanoparticles, lipid agents and polymers [72]. The advantages of using non-viral methods include ease of synthesis, high cell or tissue specificity, low immunogenicity and unlimited sequence size. However, these are often accompanied by low efficiency, high ratio of cell mortality and transient transgene expression [73]. Table 3 summarises selected studies on genetically modified MSCs.

**Table 3:** Selected studies on genetically modified MSCs

Genes involved	Enhanced effect	Key Findings	Reference
*Preclinical Studies*			
CXCR4	Increased mobility and homing effect	Modified AT-MSCs showed increased bone engraftment in mice.	[107]
HGF and VEGF	Increased angiogenesis and reduced fibrosis	Modified MSCs improved cardiac function in a porcine animal model with myocardial infarction.	[108]
HIF1A	Anti-apoptotic effect	Modified rat BM-MSCs showed improved viability and survival under hypoxia in vitro.	[109]
PDGF and HO-1	Anti-inflammatory effect	Modified AD-MSCs reduced pro-inflammatory factors and other mediators in OA in vitro as well as improved limb function and decreased pain in canine OA model.	[110]
circRNA-vgll3	Increased osteogenic differentiation and osteogenesis	Modified rat AD-MSCs enhanced new bone formation with enhanced bone volume/tissue volume, bone mineral density and trabeculae number in vivo.	[111]
BMP-2	Increased osteogenic differentiation	Modified rat BM-MSCs stimulated mitochondrial activity that led to upregulation of osteogenic differentiation-promoting PGC-1α.	[112]
PIGF-2	Promoted macrophage M2 polarization and enhanced osteogenesis	Modified mouse BM-MSCs promoted spinal fusion in mice by increasing bone formation and recruiting macrophages with M2 polarization.	[113]
PYGO1	Enhanced cardiac differentiation	Overexpressing PYGO1 boosted canonical Wnt/β-catenin signaling, promoting cardiac differentiation; inhibition of this pathway decreased specific protein levels.	[114]
E-selectin	Enhanced wound healing in ischaemic limb	Mice treated with MSCs modified with E-selectin led to faster wound healing, increased collagen deposition, and enhanced angiogenic response compared to control treatments.	[115]
*Clinical Studies*			
HGF	Symptom alleviation and improvements in lung function and imaging findings in pulmonary silicosis	• Non-randomized uncontrolled trial • Modified hBM-MSCs alleviated symptoms and improve lung functions. • Reduced serum IgG levels and increased peripheral CD4 + and CD8 + cells ratio • Reduced modular and reticulonodular lung lesions on CT scan.	[116]
HSV-Tk	Clinical stabilization of disease in advanced gastrointestinal adenocarcinoma	• TREAT-ME1 phase 1/2 study, • Treatment with modified MSCs and ganciclovir did not induce any change in tumour markers or tumor size • Treatment was well tolerated. • Disease was clinically stable.	[117]

*BM-MSCs* Bone marrow-derived mesenchymal stem cells, *BMP-2* Bone Morphogenetic Protein 2, *CXCR4* C-X-C Chemokine Receptor 4, *HGF* Hepatic Growth Factor, *HIF1A* Hypoxia Inducible Factor 1alpha, *HO-1* Haeme Oxygenase-1, *HSV-Tk* Thymidine Kinase Of The Herpes Simplex Virus, *PDGF* Platelet-Derived Growth Factor, *PGC-1α* Peroxisome proliferator-activated receptor-Gamma Coactivator-1alpha, *PIGF* Placental Growth Factor, *TREAT-ME* Tumors With Genetically Modified Autologous Mesenchymal Stromal Cells, *VEGF* Vascular Endothelial Growth Factor

#### Priming of MSCs

Biochemical and biophysical factors in the microenvironment influence the differentiation and therapeutic potentials of MSCs. Therefore, researchers have explored strategies to enhance the efficacy of MSCs by manipulating MSCs’ microenvironment. One of the strategies is priming (or preconditioning) MSCs with cytokines, hypoxia, pharmacological/chemical agents, biomaterials, specific culture conditions or a combination of various priming methods. Table 4 summarises selected studies on priming of MSCs and their enhancSince paracrine effects of MSCs ed effects, focusing on studies in the past 5 years.

**Table 4:** Selected studies on MSC priming

Priming method	Key findings	Reference
Hypoxia	Primed WJ-MSCs demonstrated increased autophagy and enhanced pro-angiogenic activity via paracrine effects.	[118]
Hypoxia	Priming increased the cell cycle life span and attenuated DNA susceptibility of chromosomal damage in human MSCs.	[119]
Specific medium + hypoxia	BM-MSCs primed with chondrogenic medium showed enhanced cartilage repair in a sheep model with cartilage defects. Ex vivo culture of chondrogenically primed BM-MSCs in hypoxic conditions before implantation improved gene expression profile of the cells but did not show consistent modulation in cartilage repair.	[120]
Specific medium + culture condition + hypoxia	hBM-MSCs that underwent long-term starvation and cultured in serum free media under hypoxic conditions demonstrated increased survival through iBiochemical and biophysical factors in the nduction of a quiescent state. hBM-MSCs showed adaptive responses by exploiting alternative source of energy and via altered expression of cell cycle regulators and PCNA.	[121]
Specific medium	AT-MSCs primed with limbal stem cell specific medium enhance wound healing in the cornea by reducing neovascularision and inflammation in a rat model.	[122]
Nicotinamide	Primed hBM-MSCs showed enhanced adipogenic, chondrogenic and osteogenic differentiation, increased in replicative lifespan, and delayed senescence.	[123]
TGFβl	Primed hBM-MSCs showed enhanced osteogenic and adipogenic differentiation via increased expression of genes related to actin cytoskeleton.	[124]
Poly I: C or IFN-γ	WJ-MSCs primed with poly I: C or IFN-α showed reduced epidermal thickness and inflammation in atopic dermatitis skin lesions in a murine model.	[125]
IL1βBiochemical and biophysical factors in the	Primed T-MSCs showed improved osteogenic differentiation and inhibition of PBMC proliferation in vitro. Primed T-MSCs contributed to enhanced bone density and bone homeostasis in ovariectomized mice with osteoporosis.	[126]
PAME	Primed rat AD-MSCs showed increased adipogenic differentiation, mediated by a pathway related to a G protein-coupled receptor.	[127]
α-synuclein	Primed MSCs exerted neuroprotective effects in mouse model of Parkinson’s disease by enhancing neuronal viability and stimulating autophagy-mediated α-synuclein modulation.	[128]
Biomaterials	Culturing hMSCs on rGO-Ti substrates enhanced osteogenic differentiation of MSCs with superior bioactivity.	[129]
Biomaterials + specific medium	Priming hMSCs with chondrogenic medium and culturing primed hMSCs using gelatin-based µRB scaffolds showed enhanced endochondral ossification in mouse model, when compared with hMSCs cultured using conventional HG scaffolds.	[130]
Biomaterials	iPSC-MSCs primed with matrix-conjugated hydrogel biomaterials showed a defined secretory profile in vitro and in vivo, which enhanced neovascularization and tubologenesis.	[131]
Biomaterials	MSCs primed with a bioengineered 3D matrix showed reduced release of osteopontin, which enhanced ex vivo expansion of HSCs co-cultured with primed MSCs.	[132]

*3D* Three Dimensional, AD/MSCs**/** *AT-MSCs* Adipose Tissue-Derived Mesenchymal Stem Cells, *HG* Gelatin Hydrogel, *IL* Interleukin, *IFN* Interferon, *iPSC-MSCs* Induced Pluripotent Stem Cell-Derived Mesenchymal Stem Cells, *PAME* Palmitic Acid Methyl Ester, *PBMC* Peripheral Blood Mononuclear Cells, *PCNA* Proliferation Cell Nuclear Antigen, *Poly I: C* Polyinosinic Polycytidylic Acid, *rGO-Ti* Reduced Graphene Oxide-Coated Titanium, *T-MSCs* Tonsil-Derived Mesenchymal Stem Cells, *µRB* microribbon, *WJ-MSCs* Wharton Jelly Derived- Mesenchymal Stem Cells

#### Harnessing and Optimizing the MSC Secretome

Since paracrine effects of MSCs are increasingly recognized as the main therapeutic pathway of action, approaches to modulate MSC secretome are currently gaining attention. Arguably, it is still the secretome consisting of soluble proteins, lipids, nucleic acids and EVs which carries much of the regenerative, immunomodulatory, and anti-inflammatory properties ascribed to MSCs [52]. Improvement of the composition and stability of this bioactive repertoire is attractive to achieve a higher therapeutic potency with addition of overcoming limitations related to donor variation, limited engraftment, and replicative senescence [53]. Various strategies have been investigated to refine the MSC secretome. Types of preconditioning approaches including hypoxia, exposure to inflammatory cytokines or pharmacological drugs provide a means to selectively enrich the secretome with factors that selectively stimulate stimulation, immunomodulation, and tissue repair [75]. In a similar fashion, genetic manipulation of MSC can steer the formation of particular therapeutic proteins or RNAs in the secretome and enhance targeted effects [32]. Culture systems such as three-dimensional (3D) spheroid culture and biomaterial scaffold culture enrich the production and bioactivity of MSC-derived EVs, substantiating their functional implication in MSC therapy [76].

The strategic placement of the secretome at the core of augmentation operations bridges the concept gap between cell-free and cell-based therapies. Emphasizing optimization of the bioactive output of MSCs can, however, underpin future development of a standardised approach to the cell-free products from MSC secretome or EVs and enhance consistency and potency of MSC-based therapies. It not only overcomes the issues of reproducibility and scalability, but also meets the registration needs and safety requirements in translation.

### Cell-Free MSC-Derived Therapeutic Approaches

Other than generating MSCs from iPSCs and enhancing MSCs’ efficacy via priming and genetic modification, another way of overcoming the challenges in cell-based therapies is the application of MSC-based cell-free derivatives. Some examples of cell-free products are conditioned medium and secretome of MSCs. Although some use the two terms interchangeably, there are slight differences between them. Conditioned medium refers to the medium in which MSCs have been cultured, including the factors secreted by MSCs into the medium. On the other hand, secretome refers to the total set of factors secreted by MSCs, which include bioactive factors like cytokines, growth factors, hormones, chemokines, cell adhesion molecules, microRNAs (miRNAs), long-coding RNAs (lncRNAs), as well as the extracellular vesicles consisting of microvesicles and exosomes [77, 78].

**Fig. 2:**
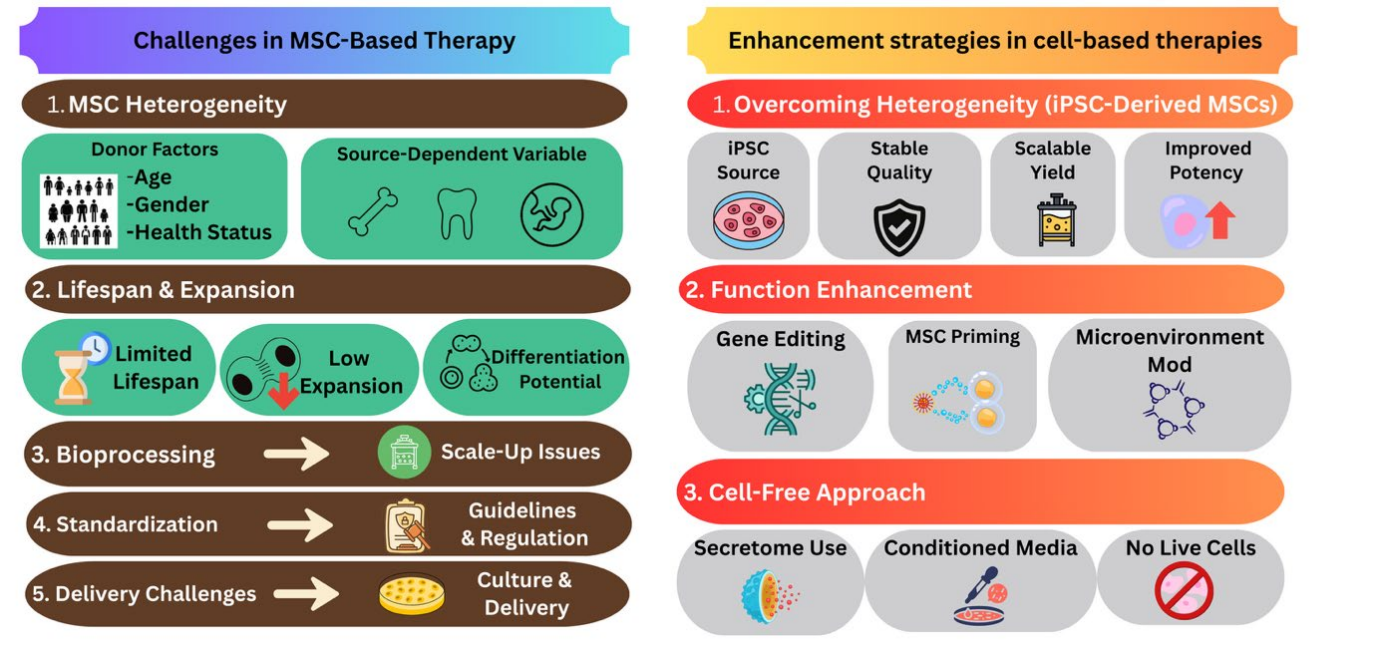
The overview of mesenchymal stem cell (MSC)-based therapies: Key challenges and the enhancement strategies

This article focusses on cell-based strategies. Therefore, a detailed discussion on the cell-free strategies is beyond the scope of this review and warrants a separate review. To this end, there is an increasing number of studies on the use of MSC conditioned medium and secretome in the published literature. For instance, conditioned medium of MSCs have been studied in wound healing [79], inflammatory [80], neurodegenerative [81] and cardiovascular diseases [82]. Research has also demonstrated the therapeutic potential of MSC secretome in ischaemic stroke [83], Parkinson’s disease [84], autoimmune diseases [85], respiratory disorders [86], etc. The overview of the mesenchymal stem cell (MSC)-based therapies highlighting the challenges and enhancement strategies are demonstrated in Fig. 2.

## Conclusions

Numerous studies have demonstrated the safety profile and therapeutic effects of MSCs from different sources in various diseases. Despite promising findings from these studies, researchers face many challenges in cell-based therapies. This is due to multiple factors contributing to MSCs heterogeneity and several restrictions on the bioprocessing of MSCs. Studies have explored various enhancement strategies to overcome challenges in cell-based therapies. However, there are not many clinical studies on these enhancement strategies. A large proportion of the published studies are preclinical studies using in vitro experiments and animal models, with some sporadic reports on studies done on human subjects. On the other hand, cell-free strategies are increasingly popular and their therapeutic potential has been demonstrated in various diseases.

Despite a growing body of research on MSC cell-free strategies, cell-based therapies using MSCs still have a place in research and treatment. This is because in some conditions, cell-to-cell contact is still necessary. For example [87], demonstrated that human amnion derived-MSCs exert their immunomodulatory effects via both cell-cell contact and soluble factors. Future research should explore the enhancement strategies for cell-based therapies in larger-scale clinical studies. The use of a combination of cell-based and cell-free strategies to overcome the limitations of cell-based therapy alone may be another option. Comparative studies on the therapeutic effects on MSCs that lead to the development of standardized protocols and guidelines at various levels of MSC bioprocessing may help overcoming the heterogeneity of MSCs.

## Data Availability

No datasets were generated or analysed during the current study.
